# The impact of subsidence on hip function: A comparative study between Proximal Femoral Nail Anti-rotation-2 and Halifax Femoral Nail in unstable trochanteric fractures

**DOI:** 10.1051/sicotj/2026027

**Published:** 2026-09-30

**Authors:** Soumya Ranjan Nayak, Amit Rastogi, Shivam Sinha, Birju Manjhi, Rahul Rai, Sushil Gandhi

**Affiliations:** 1 Department of Orthopaedics, Institute of Medical Sciences, Banaras Hindu University Varanasi 221005 Uttar Pradesh India; 2 Trauma Centre, Banaras Hindu University Varanasi 221005 Uttar Pradesh India

**Keywords:** Unstable trochanteric fractures, Intramedullary nailing, Proximal Femoral Nail Anti-rotation-2, Halifax Femoral Nail, Harris Hip Score, Subsidence

## Abstract

*Background*: Unstable trochanteric fractures pose a significant challenge in orthopedic surgery, particularly in elderly patients with poor bone quality. Achieving biomechanical stability while minimizing complications requires careful consideration of intramedullary nail design. This study aims to compare two commonly used implants – Proximal Femoral Nail Anti-rotation-2 (PFNA-2) and Halifax Femoral Nail – by evaluating their clinical, radiological, and functional outcomes, with a focus on surgical efficacy, complications, and functional recovery. *Methods*: A prospective, randomized clinical trial involving 60 elderly patients with AO/OTA 31A2 or 31A3 unstable trochanteric fractures was conducted over 1 year. Patients aged 60–80 years were randomized into two equal groups (*n* = 30 each) to receive either PFNA-2 or Halifax Nail fixation. Primary outcomes were evaluated using the Harris Hip Score (HHS) at 6 and 12 months postoperatively. Secondary outcome measures included neck-shaft angle, tip-apex distance, and screw positioning according to the Cleveland Index. Operative parameters, including surgical time, blood loss, and complications, were systematically recorded and analyzed. *Results*: Both implants demonstrated comparable efficacy regarding operative time (Halifax: 31.7 ± 5.5 vs. PFNA-2: 33.0 ± 7.9 min, *p* = 0.45) and intraoperative blood loss (Halifax: 89.5 ± 12.6 vs.PFNA-2: 97.7 ± 22.9 ml, *p* = 0.09). Halifax Nail achieved significantly superior screw positioning with 76.7% optimal center–center placement compared to 43.3% in the PFNA-2 group (*p* = 0.008). Subsidence was absent in the Halifax group but occurred in 6.7% of PFNA-2 cases. HHSs improved progressively in both groups, with Halifax showing marginally higher scores at 6 months (83.6 ± 4.3 vs.81.5 ± 6.4) and 12 months (87.4 ± 3.9 vs.85.8 ± 5.6), though differences were not statistically significant. *Discussion*: Both PFNA-2 and Halifax Nails are effective for managing unstable trochanteric fractures. The Halifax Nail’s innovative tri-wire technology™ offers superior rotational stability, optimal screw positioning, and demonstrates a trend towards reduced subsidence, making it particularly advantageous for patients with compromised bone quality. These findings support individualized implant selection based on patient factors and bone quality assessment.

## Introduction

Hip fractures are among the most severe injuries in the elderly, leading to significant morbidity, functional decline, and increased mortality. Intertrochanteric fractures account for nearly half of all hip fractures in individuals over 65 and have higher mortality and poorer functional outcomes than femoral neck fractures [1]. Unstable intertrochanteric fractures require precise surgical interventions to prevent complications like excessive collapse [2]. These injuries have higher early mortality [3], and fewer than 50% of patients regain pre-fracture walking ability within a year [4].

Fractures are classified as stable or unstable based on morphology and comminution. Unstable types (e.g., AO/OTA 31A2, 31A3) involve comminution and reverse obliquity, increasing collapse and malalignment risks [5]. Intramedullary devices like the Proximal Femoral Nail Anti-rotation (PFNA) are preferred for minimal soft tissue damage and early weight-bearing [6]. While dynamic hip screws (DHS) remain alternatives, issues like implant irritation and longer surgical time persist [7].

Challenges in managing unstable fractures include age, osteoporosis, comorbidities, and poor bone quality, increasing fixation difficulty, and implant failure [8, 9]. Surgery is standard, offering better outcomes and early mobilization [10], though complication rates remain high [11]. Osteoporosis exacerbates these risks, which PFNA and cement augmentation help mitigate [12, 13].

Intramedullary implants improve stability, reduce surgical time and blood loss, and enable early mobilization [14–16]. The PFNA-2’s helical blade compacts cancellous bone, enhancing stability and reducing cut-out risk [12, 17]. However, complications like blade migration arise from poor placement and parameters like tip-apex distance (TAD) >27 mm and neck-shaft angle (NSA) <128° [18, 19]. The Halifax Nail, with tri-wire technology™, promises enhanced rotational stability, with preliminary data suggesting superior biomechanics [20].

This prospective, randomized study compares PFNA-2 and Halifax Nail in managing unstable trochanteric fractures, evaluating operative time, blood loss, radiographic results, functional recovery, and complications.

## Materials and methods

This prospective, randomized clinical trial was conducted over 1 year at the Department of Orthopedics in a tertiary care hospital comparing PFNA-2 and Halifax Femoral Nail efficacy in treating unstable trochanteric fractures. Written informed consent was collected from all participants. Sample size was calculated based on the primary outcome measure, the Harris Hip Score (HHS) at 12 months. Assuming a standard deviation of 6, a minimum clinically important difference of 5 points in HHS, 80% power, and α of 0.05, the required sample size was 23 patients per group. Considering possible attrition, 30 patients were enrolled in each group, resulting in a total sample size of 60 patients.

Sixty patients aged 60–80 years with AO/OTA 31A2 or 31A3 fractures, eligible for surgery within 7 days, were randomized into two groups (*n* = 30 each) using computer-generated randomization.

Exclusion criteria included pathological fractures, prior proximal femur surgeries, fractures older than 1 week, and medical contraindications. Preoperative evaluation involved history-taking, physical exams, radiological assessments, and routine lab tests. Patients were prepared with fasting, antiseptic cleansing, and prophylactic antibiotics.

Surgical techniques for both implants followed similar steps involving patient positioning, fracture reduction, and fluoroscopic guidance. PFNA-2 used a helical blade, while Halifax Nail employed a tri-wire technology™ for enhanced rotational stability. Postoperative care included pain management, mobilization, imaging, and wound care, with regular follow-ups assessing pain, mobility, and healing.

Outcome measures included HHS, NSA, TAD, screw positioning, operative time, blood loss, and complications. Allocation concealment was ensured using sequentially numbered, sealed, opaque envelopes prepared by an independent staff member not involved in patient recruitment or outcome assessment. The envelopes were opened in the operating room after patient enrolment. Postoperative clinical and radiological assessments, including HHS evaluation and radiographic measurements, were performed by an independent assessor who was blinded to group allocation. Statistical analysis was performed using SPSS version 25.0. Descriptive statistics were expressed as mean ± standard deviation for continuous variables and frequencies with percentages for categorical variables. Independent t-tests compared continuous variables between groups, while chi-square or Fisher’s exact tests were employed for categorical variables. A *p*-value <0.05 was considered statistically significant.

## Results

### Demographic and clinical data

#### Age distribution

Sixty patients with a mean age of 71.5 ± 5.1 years (range: 63–80), median 72 years (IQR: 67.8–76) were included. 51.7% (31 patients) were aged 71–80, and 48.3% (29 patients) were 61–70 years. No significant age difference existed between groups: Halifax (71.3 ± 5.2) vs. PFNA-2 (71.6 ± 5.1), *p* = 0.82.

#### Gender distribution

The cohort had equal gender distribution (50% each), with 15 males and 15 females per group. No significant gender difference was observed (*p* = 1.00).

#### Mode of injury

Slip and fall was the most common cause (70%, 42 patients), followed by falls from stairs (10%), bike-related injuries (5%), and falls from chairs, hit by an auto, or bull hits (3.3% each). Injury modes were similarly distributed between groups (*p* = 0.79).

### Preoperative and operative parameters

#### Injury-to-surgery interval

The mean injury-to-surgery interval was 3.5 ± 1.2 days, median 3 days (range: 2–7). Most surgeries (41.7%) were performed within 3 days, followed by 4 days (23.3%), 2 days (16.7%), and 5 days (15%). The Halifax group had a slightly shorter mean interval (3.4 ± 0.9 vs. 3.7 ± 1.4 days), but this was not statistically significant (*p* = 0.34).

#### Blood loss

Average intraoperative blood loss was 93.6 ± 18.8 ml, median 90 ml (range: 50–200). Most patients (53.3%) had blood loss of 81–90 ml, while 36.7% had 91–100 ml. The Halifax group exhibited slightly lower mean blood loss (89.5 ± 12.6 vs. 97.7 ± 22.9 ml), but the difference was not statistically significant (*p* = 0.09).

#### Operative time

Mean operative time was 32.3 ± 6.8 minutes, median 32 min (range: 20–60). Most cases (46.7%) were completed within 20–30 min, followed by 31–40 min (41.7%). The Halifax group had a mean operative time of 31.7 ± 5.5 min vs. the PFNA-2 group, 33.0 ± 7.9 min. The difference was not statistically significant (*p* = 0.45).

### Radiological outcomes

#### NSA

Mean postoperative NSA difference was 5.2 ± 2.2 degrees, median 4 degrees (range: 2–11). Most patients (51.7%) exhibited a difference of 2–4 degrees, followed by 38.3% with 5–8 degrees, and 10% with 9–11 degrees. No significant differences between groups: Halifax 5.0 ± 2.2 vs. PFNA-2 5.3 ± 2.1 degrees (*p* = 0.64).

#### TAD

Mean TAD was 22.2 ± 2.2 mm, median 22 mm (range: 15–26). Most cases (60%) fell within a 19–23 mm range, while 33.3% had TADs of 24–26 mm, and 6.7% had 15–18 mm. Halifax group: 22.4 ± 1.8 mm vs. PFNA-2 group: 22.0 ± 2.6 mm. Differences were not statistically significant (*p* = 0.46).

#### Screw position (Cleveland Index)

Optimal center-center (CC) positioning was achieved in 60% (36 cases) of the cohort. Remaining 40% (24 cases) had a center-inferior (CI) position. The Halifax group had a significantly higher proportion of CC positions (76.7%) compared to the PFNA-2 group (43.3%, *p* = 0.008). Conversely, the PFNA-2 group had a higher proportion of CI positions (56.7%) vs. Halifax (23.3%), as shown in Table 1 and Figure 1A.

**Figure 1:**
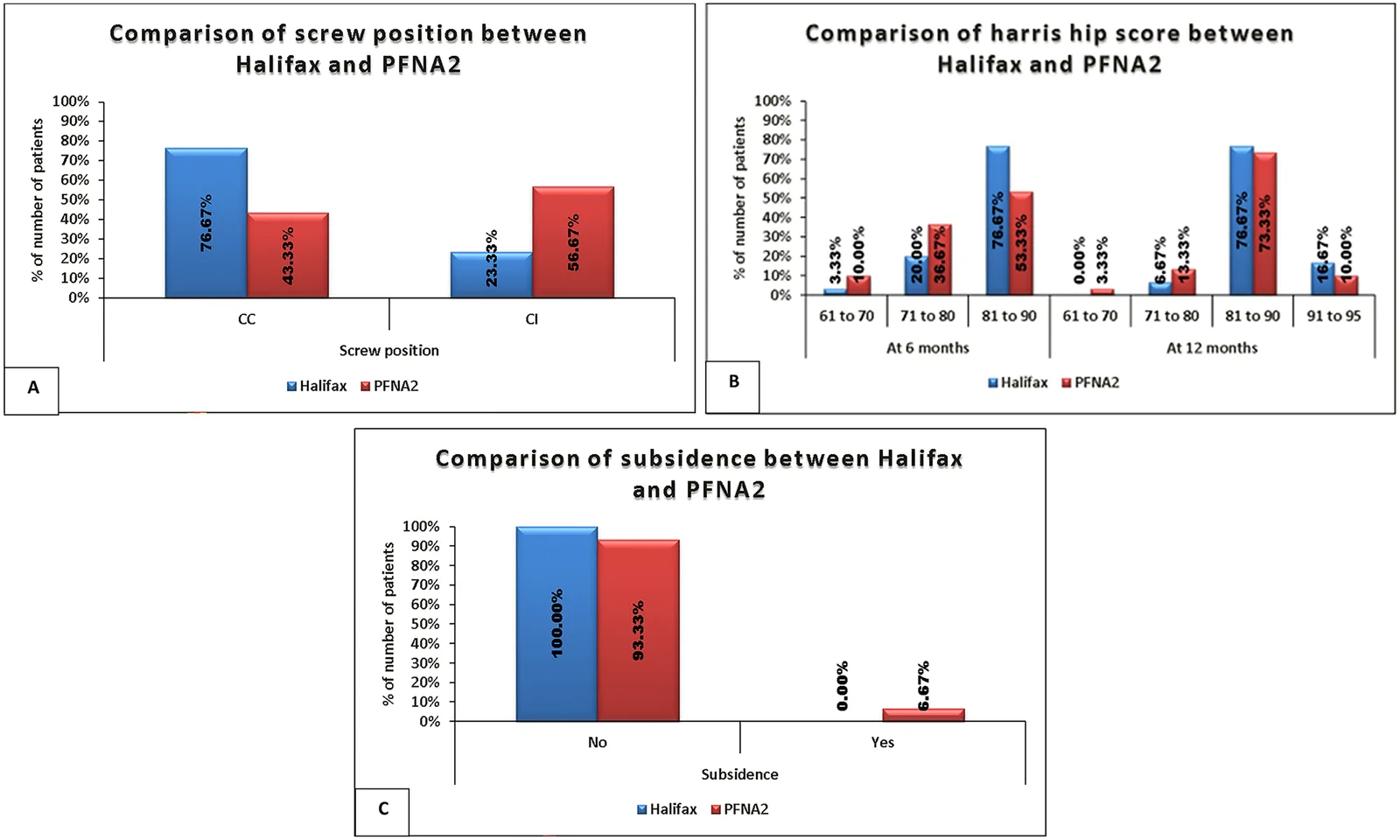
(A) Comparison of screw position between Halifax vs.PFNA2. (B): Comparison of Harris Hip Score between Halifax and PFNA2. (C): Comparison of subsidence between Halifax vs.PFNA2.

**Table 1:** Comparison of screw position between Halifax vs.PFNA2.

Screw position	Halifax (*n* = 30)	PFNA2 (*n* = 30)	Total	*p*-value
CC	23 (76.67%)	13 (43.33%)	36 (60%)	
CI	7 (23.33%)	17 (56.67%)	24 (40%)	0.008†
Total	30 (100%)	30 (100%)	60 (100%)	

^†^Chi-square test.

### Functional outcomes

HHS assessed functional recovery at 6 and 12 months postoperatively. At 6 months, mean HHS was 82.6 ± 5.5, median 84 (range: 66–90). The majority (65%) achieved scores 81–90, while 28.3% scored 71–80, and 6.7% scored 61–70. The Halifax group had slightly higher mean HHS (83.6 ± 4.3 vs. 81.5 ± 6.4), though not statistically significant (*p* = 0.14).

At 12 months, mean HHS improved to 86.6 ± 4.9, median 88 (range: 68–92). Most patients (75%) scored 81–90, with 13.3% achieving 91–95, 10% scoring 71–80, and 1.7% scoring 61–70. The Halifax group demonstrated slightly higher mean scores (87.4 ± 3.9 vs. 85.8 ± 5.6), but this was not statistically significant (*p* = 0.22).

Both groups showed progressive improvement over time. The Halifax group exhibited marginally better outcomes at both time points, yet differences were not statistically significant, indicating comparable effectiveness as shown in Table 2 and Figure 1B.

**Table 2:** Comparison of Harris Hip Score between Halifax and PFNA2.

Harris Hip Score	Halifax (*n* = 30)	PFNA2 (*n* = 30)	Total	*p*-value
*At 6 months*				
61–70	1 (3.33%)	3 (10%)	4 (6.67%)	
71–80	6 (20%)	11 (36.67%)	17 (28.33%)	0.217^*^
81–90	23 (76.67%)	16 (53.33%)	39 (65%)	
Mean ± SD	83.6 ± 4.3	81.5 ± 6.43	82.55 ± 5.52	
Median (25th–75th percentile)	84 (81.25–86.75)	83 (79.25–87.5)	84 (80–87)	0.143^‡^
Range	70–90	66–90	66–90	
*At 12 months*				
61–70	0 (0%)	1 (3.33%)	1 (1.67%)	
71–80	2 (6.67%)	4 (13.33%)	6 (10%)	0.613^*^
81–90	23 (76.67%)	22 (73.33%)	45 (75%)	
91–95	5 (16.67%)	3 (10%)	8 (13.33%)	
Mean ± SD	87.37 ± 3.93	85.8 ± 5.63	86.58 ± 4.88	
Median (25th–75th percentile)	88 (86–90)	88 (84–90)	88 (84.75–90)	0.217^‡^
Range	76–92	68–91	68–92	

^‡^Independent *t* test.

^*^Fisher’s exact test.

### Complications

Complications were minimal. Subsidence, defined as the lateral migration of the lag screw within the femoral head, was absent in the Halifax group but present in 2 patients (6.7%) in the PFNA-2 group; however, this difference was not statistically significant (*p* = 0.49) as shown in Table 3 and Figure 1C. The Halifax Nail’s innovative design offers superior screw positioning and demonstrated no subsidence in this cohort, suggesting it may be advantageous for patients with compromised bone quality. No cases of surgical site infection were reported in either group, indicating effective perioperative care and implant compatibility. No instances of non-union were noted in either group during follow-up, highlighting the effectiveness of both devices in achieving proper fracture healing.

**Table 3:** Comparison of subsidence between Halifax and PFNA2.

Subsidence	Halifax (*n* = 30)	PFNA2 (*n* = 30)	Total	*p*-value
No	30 (100%)	28 (93.33%)	58 (96.67%)	
Yes	0 (0%)	2 (6.67%)	2 (3.33%)	0.492^*^
Total	30 (100%)	30 (100%)	60 (100%)	

^*^Fisher’s exact test.

Representative radiographic examples of pre-operative fracture patterns, immediate post-operative fixation with both implant types, subsidence complications, and complete construct assessment are illustrated in Figures 2 and 3.

**Figure 2:**
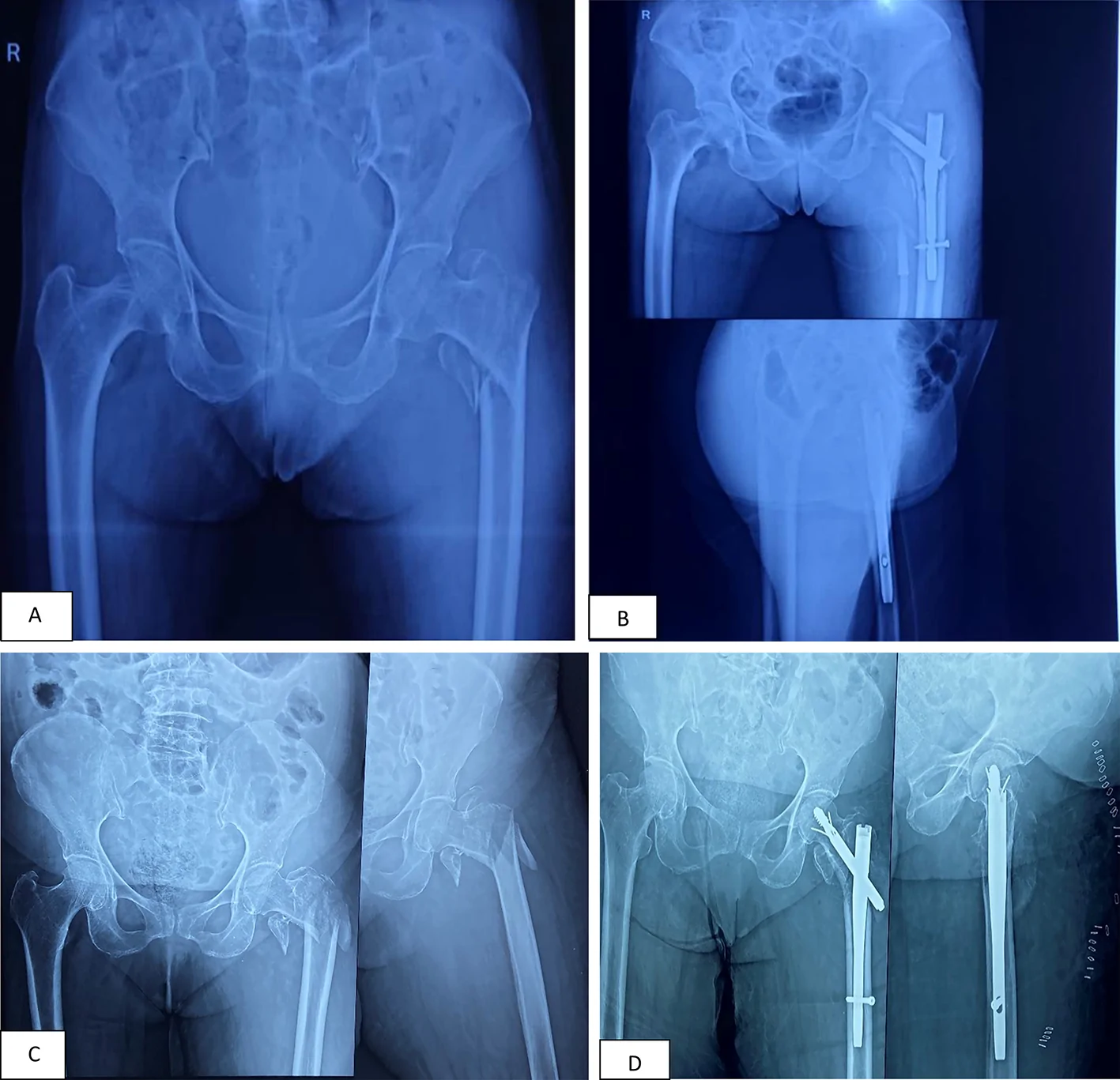
(A): PFNA-2 Pre-operative AP pelvis showing right intertrochanteric fracture. (B): PFNA-2 Post-operative radiographs (AP and lateral) demonstrating helical blade. (C): Halifax Nail Pre-operative AP pelvis showing right intertrochanteric fracture. (D): Halifax Nail Post-operative radiographs (AP and lateral) demonstrating screw via which tri-wire technology™ is employed.

**Figure 3:**
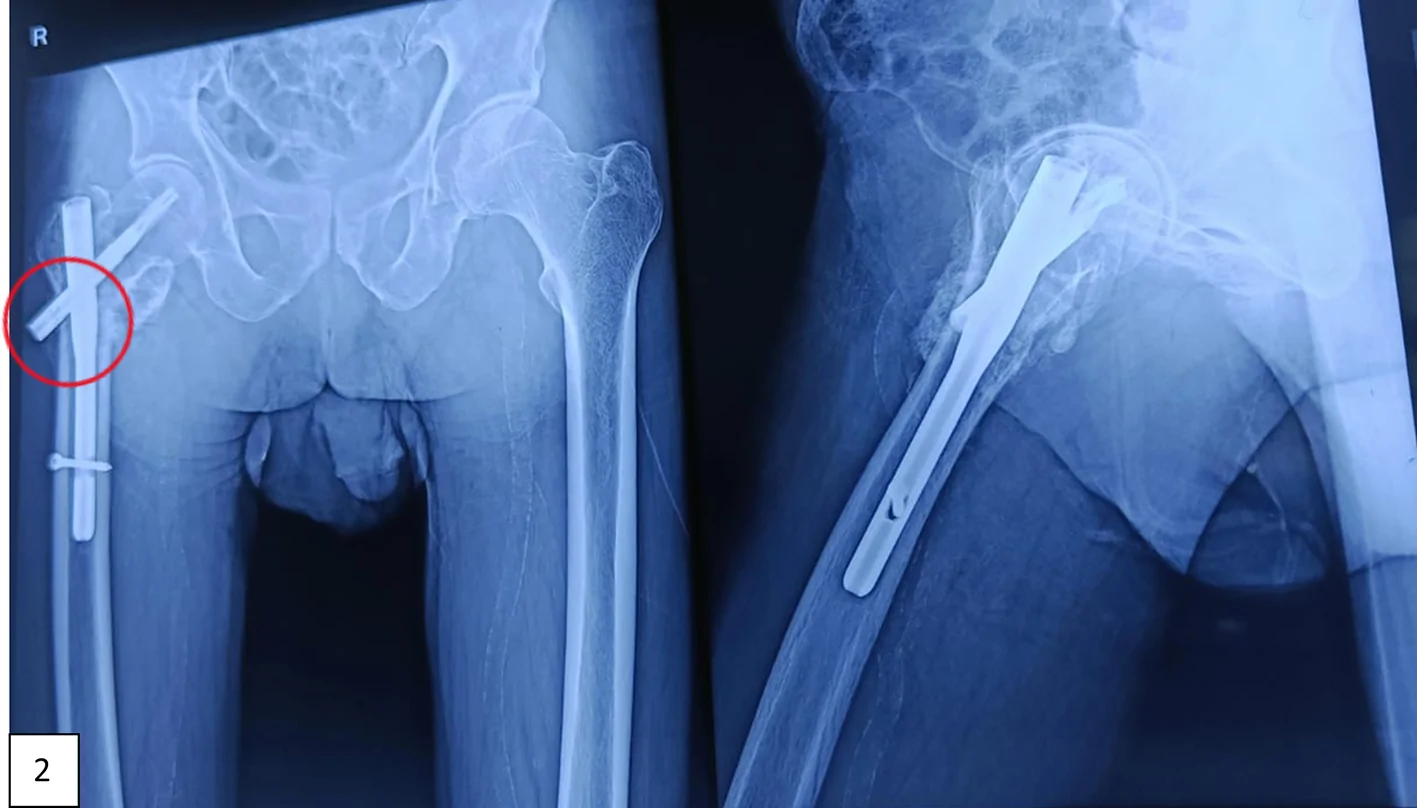
Complication of PFNA-2 fixation, AP and lateral radiographs demonstrating subsidence.

## Discussion

The management of unstable intertrochanteric fractures remains challenging, particularly among elderly patients with osteoporosis. The Halifax Femoral Nail and PFNA-2 represent advanced solutions with unique biomechanical innovations aimed at enhancing stability, minimizing complications, and improving recovery.

Both groups showed comparable age and gender distributions. The mean age (Halifax: 71.3 ± 5.2 years; PFNA-2: 71.6 ± 5.1 years) aligns with the known prevalence of intertrochanteric fractures in the elderly population. The balanced gender representation reflects broader trends reported in similar studies, where fractures are equally common among men and women over 70 years. The primary mode of injury – slip-and-fall accidents – accounted for 70% of cases, consistent with low-energy trauma typically associated with such fractures. These observations align with findings by Simmermacher et al. and Singh and Bhartiya [12, 21].

Both devices demonstrated efficient surgical timelines. Halifax Nail showed a slightly shorter injury-to-surgery interval (3.4 ± 0.9 vs. 3.7 ± 1.4 days), though not statistically significant, indicating a marginal advantage in preoperative planning. Similarly, intraoperative blood loss was marginally lower in the Halifax group (89.5 ± 12.6 vs. 97.7 ± 22.9 ml). These differences may reflect Halifax Nail’s streamlined design, reducing surgical trauma. These trends are consistent with studies by Senthilnathan et al. [22].

Halifax Nail demonstrated a slight advantage in operative time, requiring an average of 31.7 ± 5.5 vs. 33.0 ± 7.9 min for PFNA-2. Although not statistically significant, this highlights Halifax Nail’s ease of insertion. Zou et al. similarly reported longer operative times for PFNA-2 due to helical blade design requiring precise positioning [23].

Radiological outcomes highlighted device performance differences. Both maintained alignment effectively, with comparable mean NSA differences postoperatively (Halifax: 5.0 ± 2.2°; PFNA-2: 5.3 ± 2.1°). This consistency reflects biomechanical stability provided by both devices, corroborated by Knobe et al., who emphasized the importance of implant design in preserving alignment [19]. TAD showed similarly optimal values in both groups, with Halifax achieving slightly higher mean TAD (22.4 ± 1.8 mm) than PFNA-2 (22.0 ± 2.6 mm). Studies by Yam et al. stress the importance of maintaining TAD under 25 mm to minimize screw cut-out risk, achieved by both devices [18].

Halifax Nail demonstrated clear superiority in screw placement, assessed by the Cleveland Index. The higher CC rate with the Halifax Nail likely reflects implant biomechanics rather than surgeon preference. PFNA-2 blades are often placed CI to improve purchase in the denser inferior cortex and reduce migration risk without a locking mechanism. In contrast, the Halifax lag screw is secured with a locking set screw and requires central alignment for its tri-wire technology™, thereby favoring CC placement. Optimal CC positioning was achieved in 76.7% of Halifax cases vs. 43.3% of PFNA-2 cases, a statistically significant difference (*p* = 0.008). This finding is consistent with Halifax Nail’s innovative tri-wire technology™, enhancing precision during implantation. By contrast, PFNA-2’s helical blade design has been associated with higher rates of suboptimal positioning, contributing to complications such as rotational instability and screw migration, as noted by Simmermacher et al. [12].

Functional outcomes, measured using the HHS, showed significant improvements in both groups over time. At 6 and 12 months, the Halifax group consistently achieved slightly higher mean HHS scores than PFNA-2, reflecting a trend toward superior functional recovery. While differences were not statistically significant, they align with findings by Schipper et al., who reported similarly effective functional outcomes for PFNA-2 and other intramedullary devices [24].

Complication rates provided further insights into relative advantages. Subsidence, defined as the lateral migration of the lag screw, was absent in the Halifax group but observed in 6.7% of PFNA-2 cases. This highlights Halifax Nail’s advantage in preventing screw migration, likely due to the patented set screw mechanism, and hence demonstrating a potential for reduced subsidence. By contrast, PFNA-2’s helical blade, while effective in achieving cancellous bone compression, has been associated with higher migration and subsidence risks, particularly in osteoporotic patients. These trends align with studies by Kammerlander et al., who identified subsidence as a common limitation of PFNA-2 in patients with compromised bone quality [17].

### Limitations

Small sample size, single-center study.

## Conclusion

Both PFNA-2 and Halifax Nail are effective for managing unstable trochanteric fractures. The Halifax Nail’s innovative design offers superior screw positioning and demonstrates a reduced trend towards subsidence, making it advantageous for patients with compromised bone quality. The Halifax Nail achieved significantly superior CC screw positioning according to the Cleveland Index, which may contribute to improved biomechanical stability. These findings support individualized implant selection based on patient factors and bone quality assessment. Further research is needed to confirm these findings with longer follow-up periods.

## Data Availability

Data from the study is available on request from the corresponding author due to privacy restrictions.
